# Statistical analysis of mutant allele frequency level of circulating cell-free DNA and blood cells in healthy individuals

**DOI:** 10.1038/s41598-017-06106-1

**Published:** 2017-08-08

**Authors:** Ligang Xia, Zhoufang Li, Bo Zhou, Geng Tian, Lidong Zeng, Hongyu Dai, Xiaohua Li, Chaoyu Liu, Shixin Lu, Feiyue Xu, Xiaonian Tu, Fang Deng, Yuancai Xie, Weiren Huang, Jiankui He

**Affiliations:** 1 Department of Gastrointestinal Surgery, Shenzhen People’s Hospital, the Second Clinical Medical College of Jinan University, Shenzhen, 518020 China; 2 Department of Biology, South University of Science and Technology of China, Shenzhen, 518055 China; 3 Department of Oncology, Shenzhen Second People’s Hospital, Shenzhen, China; 4 Direct Genomics Co., Ltd., Shenzhen, Guangdong, 518055 China; 5 Shenzhen GeneHealth Bio Tech Co., Ltd., Shenzhen, Guangdong 518053 China; 6 Department of Clinical Laboratory, Anhui Provincial Cancer Hospital, West Branch of Anhui Provincial Hospital, Anhui Medical University, Hefei, China; 7 Thoracic Department, Peking University Shenzhen Hospital, Shenzhen, China; 8 Surgical Department of Urology, Shenzhen Second People’s Hospital, Shenzhen, China

## Abstract

Cell-free DNA (cfDNA) in plasma has emerged as a potential important biomarker in clinical diagnostics, particularly in cancer. However, somatic mutations are also commonly found in healthy individuals, which interfere with the effectiveness for cancer diagnostics. This study examined the background somatic mutations in white blood cells (WBC) and cfDNA in healthy controls based on sequencing data from 821 non-cancer individuals and several cancer samples with the aim of understanding the patterns of mutations detected in cfDNA. We determined the mutation allele frequencies in both WBC and cfDNA using a panel of 50 cancer-associated genes that covers 20 K-nucleotide region and ultra-deep sequencing with average depth >40000-fold. Our results showed that most of the mutations in cfDNA were highly correlated to WBC. We also observed that the *NPM1* gene was the most frequently mutated gene in both WBC and cfDNA. Our study highlighted the importance of sequencing both cfDNA and WBC to improve the sensitivity and accuracy for calling cancer-related mutations from circulating tumour DNA, and shedded light on developing a strategy for early cancer diagnosis by cfDNA sequencing.

## Introduction

A high percentage of cancers are diagnosed at advanced stages, resulting in the failure of curative treatment and poor quality of life for patients^1, 2^. Thus, the identification of diagnostic markers would enable earlier diagnosis and more successful treatment for cancer patients. Cell-free DNA (cfDNA) in the blood circulation and circulating tumour DNA (ctDNA) in the plasma in cancer patients have emerged as potential important biomarkers for cancer monitoring and treatment^3^. Multiple groups recently reported that the levels of plasma ctDNA were elevated in cancers^4, 5^. Previous studies proposed that cfDNA could be used as a non-invasive, sensitive tool for early diagnosis of cancer^6–10^.

The circulating cfDNA is a mixture of DNA from blood cells, viruses, solid organs and many other sources. In healthy individuals, over 90% of cfDNA is from debris of blood cells. However, in cancer patients, tumour-related ctDNA comprises 0.1–0.01% of the plasma cfDNA, which means that accurate detection of ctDNA for cancer early diagnosis must involve the removal of the background of somatic mutations that originate from blood cells^11–13^. However, there is limited knowledge available for the somatic mutations in normal cells, particularly in WBCs, which are the main contributors for the background mutations. Human genomic mutations occur at a rate of approximately 2.5 × 10^8^ per base per cell generation^14^. The Campbell group studied the background mutation spectrum of skin samples and focused on 74 cancer genes in normal skin samples from four individuals; their results proved that somatic mutations accumulate in normal cells, although the mechanisms were poorly understood^15^. Together this indicates that a better understanding of the baseline spectrum of somatic mutations in healthy individuals is urgently required before the detection of ctDNA can be useful in early cancer diagnosis.

With the dramatically decreased cost of next-generation sequencing (NGS) in recent years, it is now possible to screen a large number of individuals at ultra-deep sequencing depths to identify cancer-related mutations^16–19^. The development of molecular technology to suppress the technique noise also makes it possible to pinpoint the mutations, fusions and copy number variations related to cancer in the ultra-low concentration of cfDNA. Both academic research groups and industry players are chasing the pan-cancer screening by a simple blood draw. However, the reliable and accurate application of ctDNA detection requires better understanding of background somatic information in healthy individuals.

Our studies were designed to reveal the background somatic mutations in cfDNA by ultra-deep sequencing for 50 cancer-associated genes in 1134 non-cancer samples and several cancer samples, providing critical information of the baseline profile of somatic mutations in healthy individuals, which will fill the gap in early cancer diagnosis and personalized cancer therapy.

## Results

### Validating the detecting sensitivity using the standard reference and tumor samples

**Figure 1:**
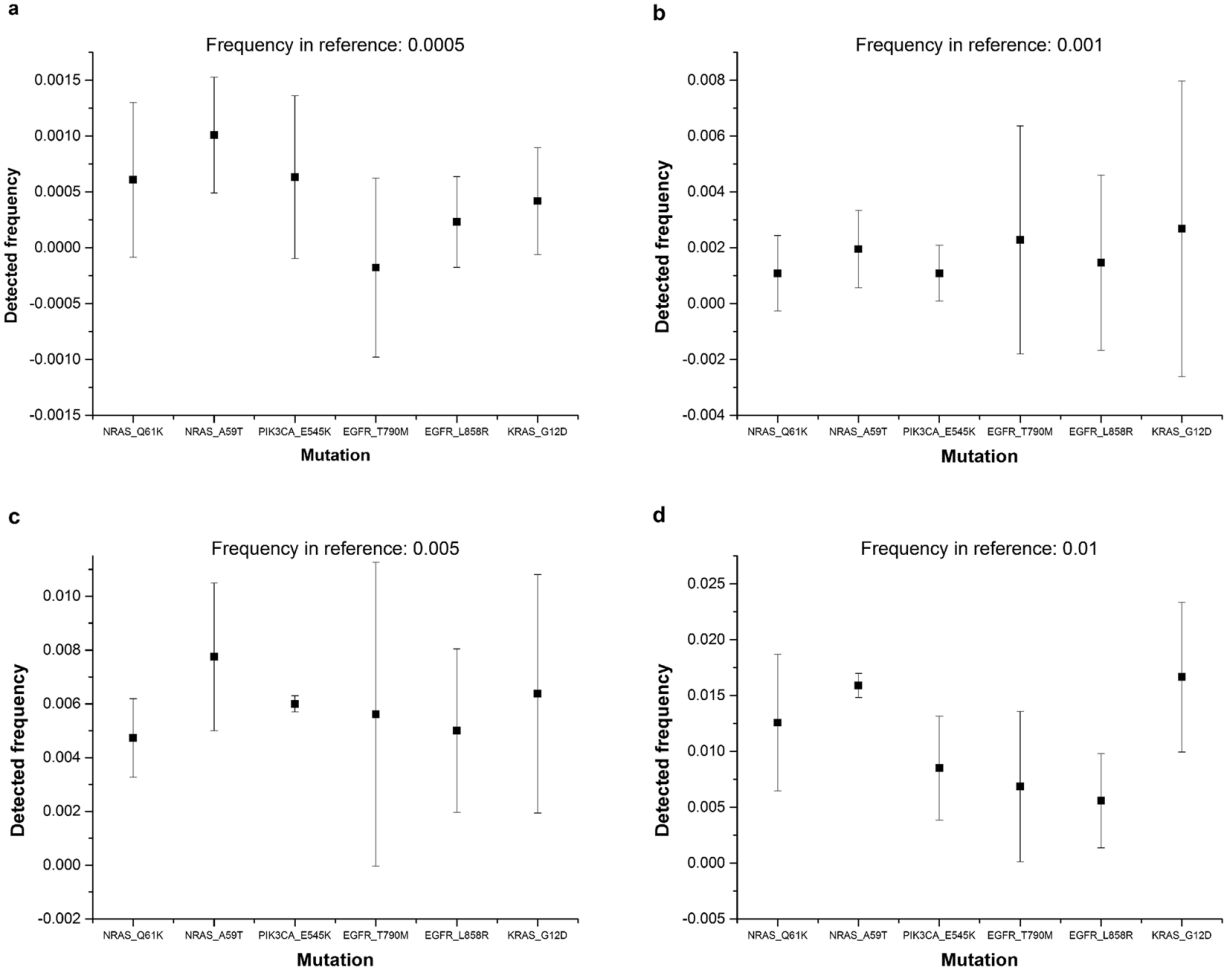
Sensitivity test using Horizon Reference Standard for cfDNA. The reference was manufactured from engineered human cancer cell lines with an allele frequency of 0.0005, 0.001, 0.005, and 0.01. We performed deep sequencing and calculated the allele frequency detected by sequencing in five repeated experiments. Error bars are standard errors.

To evaluate the sensitivity and precision of our method used in cfDNA analysis, we firstly tested the method by sequencing the Horizon Standard Reference libraries. The reference libraries cover six hotspot mutations in *EGFR*, *NRAS*, *PIK3CA* and *KRAS* genes. The average size of the reference genome is around 160 bp, mimicking the plasma cfDNA that we derived from blood plasma. We examined the sensitivity and precision of this cfDNA reference standard using our sample and library preparation method and tested the sensitivity of the method at variant allele frequencies of 0.0005, 0.001, 0.005 and 0.01. We sequenced the library at an average depth of >30000-fold coverage. The results demonstrated that our method could detect the majority of variant alleles at a frequency of 0.001; however, the average allele frequency ranged from 0.001 to 0.003, indicating substantial stochastic fluctuation (Fig. 1). When the allele frequency in the reference was 0.01, the performance of our method was much better and the stochastic fluctuation was much smaller (Supplementary Fig. S1 and Supplementary Table S1). Therefore, our method may be used to detect the variant alleles qualitatively at a frequency of 0.001 and quantitatively at a frequency of 0.01.

We also demonstrated the possibility of using mutation in cfDNA as the diagnostic marker for revealing the real mutation in tumor tissue. We recruited five new patients with colon cancer and extracted the genomic DNA of WBC, cfDNA and genomic DNA from tumor tissue for each patient. The mutation information of five above patients was summarized in Supplementary Table S2. Then (1) we checked the mutations in two tumor sections collected at different positions from the same solid tumor mass of the same patient; (2) we checked the mutations of cfDNA in blood sample of the same patient. In total, we detected 25 mutations and all these mutations obtained were these filtered by WBC. Among the 25 mutations, (1) there were 6 mutations shared between tumor section 1 and tumor section 2; (2) there were 3 mutations shared between cfDNA and tumor section 1 while 4 mutations shared between cfDNA and tumor section 2. In summary, we do admit that cfDNA may not 100% represent the condition of tumor progress, but it is undoubtedly a powerful tool in clinical practice to help diagnose cancer and evaluate the treatment effect to some extent, particularly when the tumor tissue biopsy is hardly feasible.

### The mutations detected in cfDNA are highly correlated with mutations in WBCs

**Figure 2:**
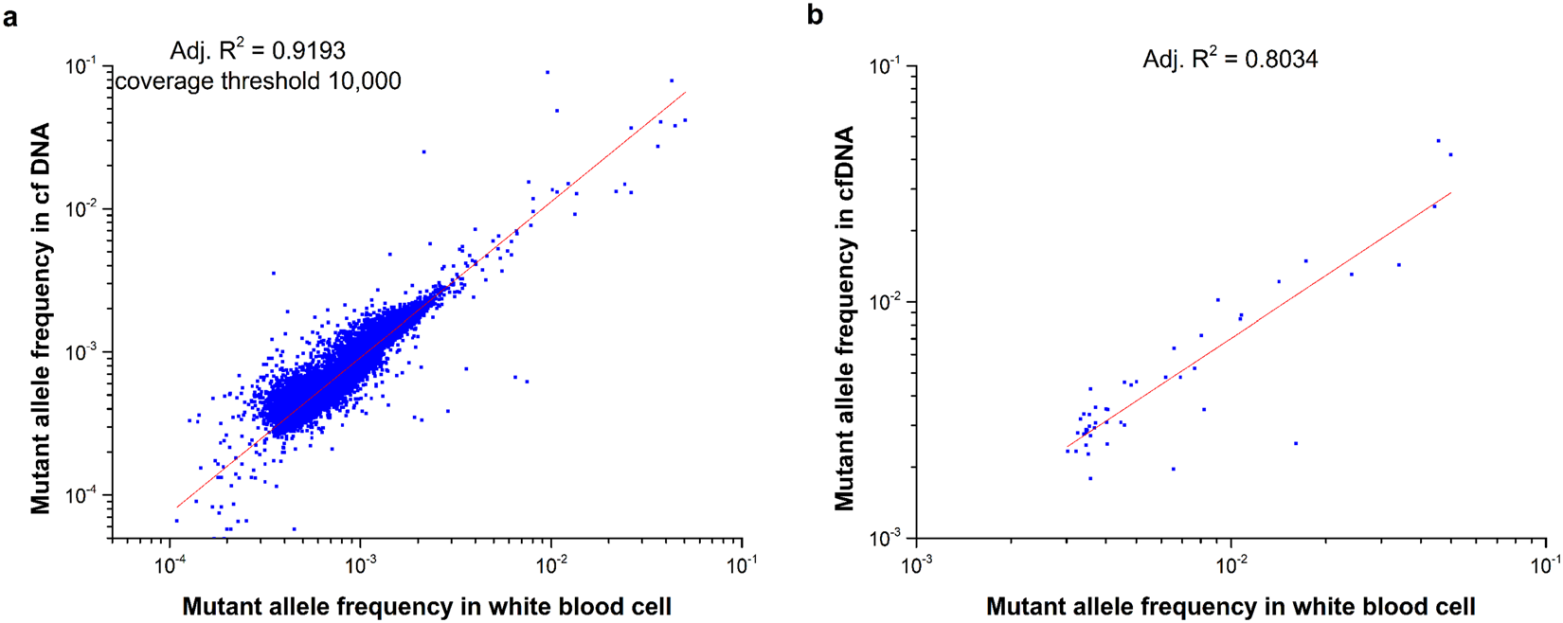
Correlation study of mutant allele frequency in ctDNA and white blood cells. (**a**) We analysed the correlation of mutant allele frequency between cfDNA and genomic DNA of white blood cells. A total of 309 paired samples were examined. Each dot represents the mutant allele frequency of one position in WBC and its corresponding cfDNA, averaged for 309 samples. (**b**) Correlation study of one individual. Only the total sequencing depth larger than 10000× and mutant allele frequency larger than 0.3% in both white blood cells and cfDNA were selected in this analysis.

We performed ultra-deep target sequencing on 50 cancer-associated genes for both plasma DNA (cfDNA) and blood cell DNA (Supplementary Fig. S2). We analysed the correlation of the mutant allele frequency in 309 WBC samples and the corresponding cfDNA. In Fig. 2a, each dot represents the average mutant allele frequency of one position in both WBC and cfDNA. Pearson’s correlation study suggested that the mutant allele frequency of cfDNA and WBC is highly correlated (adjusted R^2^ = 0.92) (Fig. 2a and Supplementary Fig. S3a–c). We plotted the data from only one individual and found that the mutant allele frequency of WBC compared with cfDNA in one individual sample was also highly correlated, with R^2^ = 0.80 (Fig. 2b). The somatic mutations in the blood cells significantly contribute to mutations in cfDNA. These results also highlight the importance of sequencing both cfDNA and blood cells to remove the background mutations contributed by blood cells.

### Reproducibility

**Figure 3:**
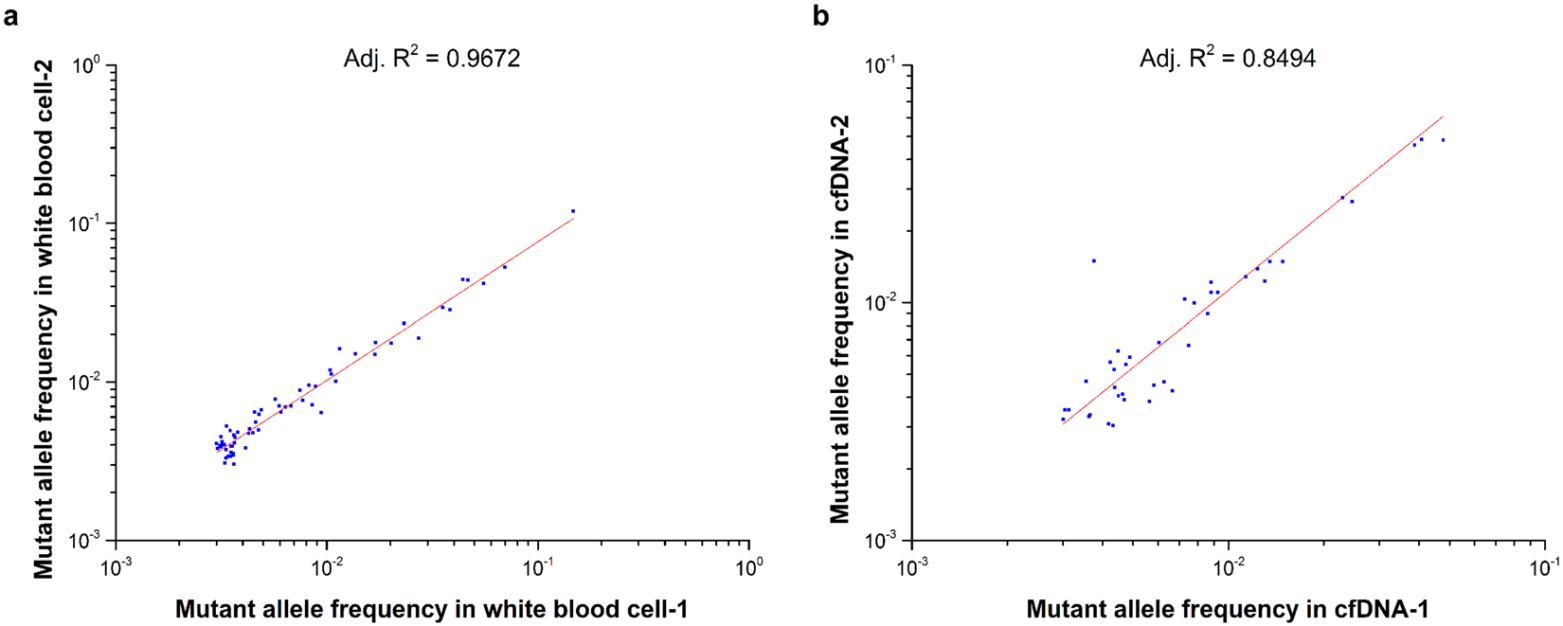
Reproducibility validation. We compared the sequencing information of two replicate WBC samples (**a**) and cfDNA samples (**b**) to evaluate the reproducibility of the methods. Only a total sequencing depth larger than 10000× and mutant allele frequency larger than 0.003 were included in the correlation study.

One of the key issues to be addressed is to distinguish the true biological variations from experimental noise or errors. Two technical replicates were performed for both WBC and cfDNA samples from the same individual, and then we compared the mutant allele frequency in the two WBC samples and the two cfDNA samples. The reproducibility validation experiment in the two WBC samples was used to estimate the PCR error rate and sequencing errors in our study cohort. Our data showed that the mutant allele frequency in the WBC samples were highly correlated (Adj R^2^ = 0.9672), which suggested the mutations with frequency >0.3% were highly reproducible (Fig. 3a and Supplementary Fig. S3). The two cfDNA samples also showed good correlations (Adj R^2^ = 0.85), but were not as good as the WBC samples (Fig. 3b). The lower correlation of the cfDNA replicates may arise from both biological and technique variations. For example, cfDNA is more complex in composition and comprises debris of different tissues, and each subtype may contain a very small amount of DNA. The tiny amount of cfDNA for experiments could increase the difficulty for effective amplification, and therefore introduce technique variations during the amplification process.

### *NPM1* is the most frequently mutated gene

**Figure 4:**
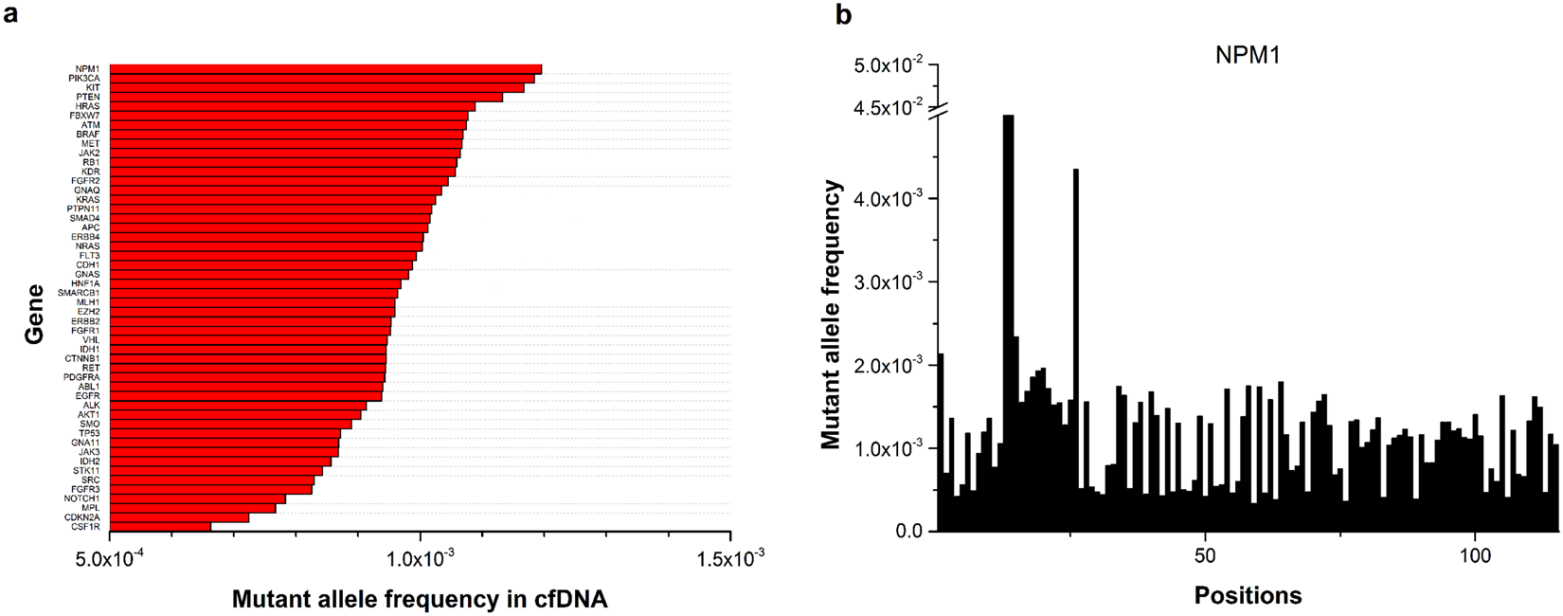
Ranking of the average mutant allele frequency of 50 cancer-associated genes. (**a**) Mutant allele frequency per position for the 50 cancer-associated genes. (**b**) The mutant allele frequency of each position in the *NPM1* gene. Here, only one amplicon was designed for *NPM1* in our study.

The mutation frequencies of 50 genes were analysed and ranked by comparing 309 cfDNA samples with average reads depth more than 10000X (Fig. 4a). For example, mutation frequency for gene A F_A_ = (F1 + F2 + … + Fi + … + Fn)/n, (n: length of the amplicon of gene A in this study, Fi: total frequency of non reference alleles at position i (i = 1 … n). The top four mutated genes were *NPM1*, *PI3K*, *KIT* and *PTEN* genes (Fig. 4b and Supplementary Fig. S4a–c). The average mutant allele frequency of the *NPM1* gene in each nucleotide was up to 0.12%. This observation was detected in both the cfDNA and WBC (Supplementary Fig. S4d), suggesting that the major origin of *NPM1* mutations was from blood cells. This result suggests that caution needs to be taken for these blood prone mutations when using cfDNA as a diagnostic marker.

In order to show the profile of hotspot mutations in cfDNA, we selected 775 cfDNA samples from total 821 cfDNA dataset that with good sequencing quality and calculated the mutation frequency for each gene) (Supplementary Figure S5). To increase the confidence of the called mutations, we here define the mutation as the variant allele frequency larger than 1% and the average depth more than 5000x for demonstration. The ranking for the top mutated genes was slightly different from that when we calculated all the positions in the selected amplicons. The number of hotspot mutations detected in each individual for all 1134 non-cancer samples were summarized in Supplementary Table S3. We also showed one example of the mutant allele frequency of all 2800 hotspots in both WBC and cfDNA (Supplementary Table S4).

## Discussion

In recent years, cfDNA and ctDNA has emerged as the research frontier of non-invasive cancer biomarkers for the detection, monitoring and treatment of cancer^20–25^. However, several challenges have prevented the use of cfDNA and ctDNA in early diagnosis strategies for cancer.

The first challenge is the system errors derived both from the PCR and the sequencing. The ctDNAs are released to the peripheral blood in the very beginning of tumour initiation, far before the tumour can be detected by either CT or other clinical tools. However, in the very early stage of tumours, the amount of ctDNA is as low as 0.1–0.01%. The sequencing alone may introduce ~0.1% error by Illumina sequencing chemistry. Several groups have proposed to use digital barcoding of every molecule to reduce the errors of PCR and sequencing^26^. We also pursued digital barcoding and the results will be published in upcoming articles.

The second challenge is distinguishing the tumour mutations from the background mutations from WBC. More than 90% of cfDNA are from blood cells, and we also observed that mutations in cfDNA were highly correlated to those in the WBC. Therefore, the majority of mutations detected in cfDNA were the background mutations from WBC. The cfDNA also contains many other sources of DNA, and its composition and origin varies by individual and by different disease states. It is critical to perform systematic analysis of WBC somatic mutations and investigate the origin of cfDNA to understand all factors that may contribute to the mutation spectrum of cfDNA. In our study, we demonstrated the importance of sequencing both cfDNA and WBC for every individual.

Somatic mutations in healthy patients are very prevalent, with an average mutation number of around 2–6 mutations/1 M bases^6, 15, 26^. There is little information available to determine these biologically significant variations in cfDNA. This study revealed the baseline mutation spectrum in WBC and cfDNA of healthy patients and can help fill the gaps for the establishing early cancer diagnosis strategies. We observed an interesting pattern in WBC, in that the *NPM1* gene was mutated at a very high rate. The average mutant allele frequency for the *NPM1* gene in all 1134 samples of healthy controls was as high as 0.12%. The *NPM1* mutations behave as gatekeepers for leukaemia and have been reported to be important genetic contributors in multiple types of leukaemia^27^. Surprisingly, the *TP53* gene was not the most frequently mutated gene in cfDNA samples. This observation can be explained by fact that *TP53* mutations are more frequent in solid organ tumours, while the cfDNA is mainly from blood cells. Therefore, we need to carefully calibrate the background mutations in WBC and cfDNA of healthy individuals to reduce a false positive rate during early cancer diagnosis.

Our study can help define the threshold of mutation detection of ctDNA by removing the background mutations in WBC and cfDNA in a healthy population. In the future, three aspects should be further improved: (1) a larger sample size is required to refine the baseline spectrum; (2) experimental methods that can increase the detection limit and remove technique noise are highly desired; and (3) an efficient bioinformatics pipeline needs to be developed to remove the noise and increase the accuracy and sensitivity in the mutation detection of ctDNA.

## Methods

### Ethics

This study was approved by the Second People’s Hospital of Shenzhen (The First Affiliated Hospital of Shenzhen). All the experiments were performed in accordance with guidelines and regulations of the Second People’s Hospital of Shenzhen. Written consent was obtained from each participant and all analyses were performed anonymously.

### Patients

A total 1149 samples were included in this study (Supplementary Table S5), which included 821 cfDNA samples and 313 WBC samples from non-cancer individuals (Only 309 WBC sequencing data, 4 are lost during sample preparation) and other 15 samples from 5 cancer patients. The sample group comprised 56.6% females and 43.4% males. The participant ages ranged from 16 to 86 years, with a median of 42 years old. Our analysis of the patient clinical information revealed no significant genetic diversity in the samples. The statistical analysis of sample information is summarized in Table 1.

**Table 1:** Summary of characteristics of 821 non-cancer individuals.

Age years	Parameter value
Mean (SD)	43.1 (12.8)
Median (range)	42.5 (16–86)
10–19 n (%)	2 (0.2)
20–29 n (%)	128 (15.6)
30–39 n (%)	233 (28.4)
40–49 n (%)	194 (23.7)
50–59 n (%)	158 (19.3)
60–69 n (%)	87 (10.6)
70–79 n (%)	17 (2.1)
80–89 n (%)	1 (0.1)
**Gender**	
Female n (%)	464 (56.6)
Male n (%)	356 (43.4)
**Smoking**	
Female n (%)	62 (7.6)
Male n (%)	149 (18.1)
**Family tumor history**	
single parent n (%)	81 (9.9)
both parents n (%)	2 (0.2)

### Blood plasma isolation

For each sample, 10 ml of blood in a cell-free DNA collection tube (VanGenes, Xiamen, China) was collected and then centrifuged at 1600 g for 10 min at 4 °C to roughly separate the sample into plasma and blood cells. The upper phase was then transferred into a new tube, leaving around 8 mm of “buffering” layer from the buffy coat after the centrifugation and avoid contaminating the plasma layer by blood or cell debris. The plasma was further centrifuged at 16000 g for another 10 min to remove the cell debris. The upper clear layer was then aliquoted into 2-ml tubes, clearly labelled with the patients’ identity and immediately stored at −80 °C for cfDNA extraction.

### Extraction of cfDNA

Each extraction of cfDNA was performed from 1 ml of plasma. Extraction of cfDNA was conducted using the E.Z.N. A Circulating DNA Kit (Omega Bio-tek, GA, USA). Genomic DNA of WBCs was extracted by the Qiagen DNA mini kit (Qiagen, Hilden, Germany). The concentration of extracted DNA was measured using the Qubit 2.0, dsDNA high-sensitivity assay (Life Technologies, Carlsbad, CA). All methods were performed according the manufacturers’ instructions.

### Spike in control

The sensitivity and precision of the current method were evaluated by Horizon’s Partners Spike-in control (Horizon, Cambridge, UK) using our 207-pair primer set. Briefly, we performed a serial dilution (from 0.0005 to 1) using the wild-type reference genome and the provided reference standard. We performed multiplex PCR, and then library construction and sequenced on the HiseqX10 (Illumina, CA, USA). Six reference variants were included in our primer set: EGFR (L858R, T790M), KRAS (G12D), NRAS (Q61K, A59T) and PIK3CA (E545K). All other primers were used as the background.

### Multiplex PCR and sequencing library construction

We developed a multiplex PCR method to enrich cancer-associated genes. Fifty cancer-associated genes were included and were amplified by 207 amplicons and covered a 22027 bp region. A total 2800 mutations were defined as hotspot mutations according to Mayo CliWe used 10 ng of DNA per sample for amplicon production by multiplex PCR. The resulting multiplex PCR reaction pool was further used in sequencing library preparation using the reagents provided in the same kit following the manual’s instructions. The sequencing library was then sent to WuxiNextCODE on the Illumina Hiseq X10 platform (Illumina, Beghelli, CA, USA).

### Data filtering and analysis

We performed ultra-deep target sequencing on 50 cancer-associated genes for both cfDNA and WBC DNA. For each sample, the average sequencing depth was 40000× . The sequencing data was first mapped to the human reference genome (human genome build19; hg19) by Burrows–Wheeler transformation (BWA, Version: 0.7.5a-r405) software package, converted to mpileup format for downstream analysis. We set two filtering criteria to filter the reads: (1) read sequences with mutant allele frequency higher than 5% in a single reads were deleted; and (2) bases with base quality lower than 30 were deleted. Loci with sequencing depth less than 10000× were removed for further analysis.

### Mutant allele frequency in cfDNA and WBCs in the population and in individuals

We analysed the correlation of the mutant allele frequency between cfDNA and genomic DNA of WBCs. The mutant allele frequency was defined as mutant allele frequency divided by total reads covering the locus. For example, for a particular position, the total sequencing depth is 10000; we obtain 9990 for A, 3 for C, 5 for G, and 2 for T, and thus the mutant allele frequency for this position is (3 + 5 + 2)/10000 = 1/1000. Among the samples from 821 individuals, we only used 309 pairs of samples for which both cfDNA and WBCs were available from the same person (collected at the same time). A total of 22027 nucleotides in 50 genes were included in this study. We calculated the average mutant allele frequency of each position in 309 individuals in WBCs and cfDNA. We removed the positions with mutant allele frequency higher than 0.1.

### Reproducibility evaluation

To validate the reproducibility of our methods, we drew blood from an individual, spilt the sample in half, and performed multiplex PCR and sequencing independently. By comparing the sequencing data of the two replicates in WBC samples, we were able to evaluate the stability of the methods and remove background noise. Positions with a sequencing depth lower than 10000× and mutant allele frequency larger than 0.003 were excluded in the correlation study.

### Data Availability

The datasets generated during and/or analysed during the current study are available in Sequence Read Archive with accession number SRP105315. The datasets are also available from the corresponding author on reasonable request.

## Electronic supplementary material

Supplementary information

Table S1

Table S2

Table S3

Table S4

Table S5
