# Supplementary material for: Statistical analysis of mutant allele frequency level of circulating cell-free DNA and blood cells in healthy individuals
Source: Sci Rep. 2017 Aug 8;7:7526. doi: 10.1038/s41598-017-06106-1 (PMC5548860; doi:10.1038/s41598-017-06106-1)
Supplement: Supplementary file 1 — Supplementary information [file 41598_2017_6106_MOESM1_ESM.doc]

**Supplementary Information**

**Statistical analysis of mutant allele frequency level of circulating cell-free DNA and blood cells in healthy individuals**

Ligang Xia^1#^, Zhoufang Li^2#^, Bo Zhou^2#^, Geng Tian^3#^, Lidong Zeng^4^, Hongyu Dai^5^, Xiaohua Li^5^, Chaoyu Liu^5^, Shixin Lu^2^, Feiyue Xu^5^, Xiaonian Tu^5^, Fang Deng^6^, Yuancai Xie^7^, Weiren Huang^8^, Jiankui He^2,4,5^*

^1^Department of Gastrointestinal Surgery, ^3^Department of Oncology, ^6^Surgical Department of Urology, Shenzhen People’s Hospital, the Second Clinical Medical College of Jinan University, Shenzhen 518020, China

^2^Department of Biology, South University of Science and Technology of China, Shenzhen 518055, China

^3^Department of Oncology, Shenzhen Second People’s Hospital, Shenzhen, China

^4^Direct Genomics Co., Ltd., Shenzhen, Guangdong 518055, China

^5^Shenzhen GeneHealth Bio Tech Co., Ltd., Shenzhen, Guangdong 518053, China

^6^Department of Clinical Laboratory, Anhui Provincial Cancer Hospital, West Branch of

Anhui Provincial Hospital, Anhui Medical University, Hefei, China

^7^Thoracic Department, Peking University Shenzhen Hospital, Shenzhen, China

^8^Surgical Department of Urology, Shenzhen Second People’s Hospital, Shenzhen, China

#These authors equally contributed to the manuscript.

*Corresponding author: Jiankui He, [hejk@sustc.edu.cn](mailto:hejk@sustc.edu.cn)

**ctDNA and FFPE sample sequencing from cancer patients**

We recruited five new patients with colon cancer and extracted the cfDNA, genomic DNA of WBC and genomic DNA from tumor tissue for each patient. The mutation information of five above patients is summarized in Table S2. We compared the mutations in two tumor sections collected at different positions from the same solid tumor mass of the same patient. Meanwhile, we compared the mutations of cfDNA in blood sample with the mutations in the tumor sections of the same patient.

In total, we detected 25 mutations in two tumor sections. Among the 25 mutations, 6 mutations are shared between tumor section 1 and tumor section 2. There are 3 mutations shared between cfDNA and tumor section 1, while 4 mutations are shared between cfDNA and tumor section 2.


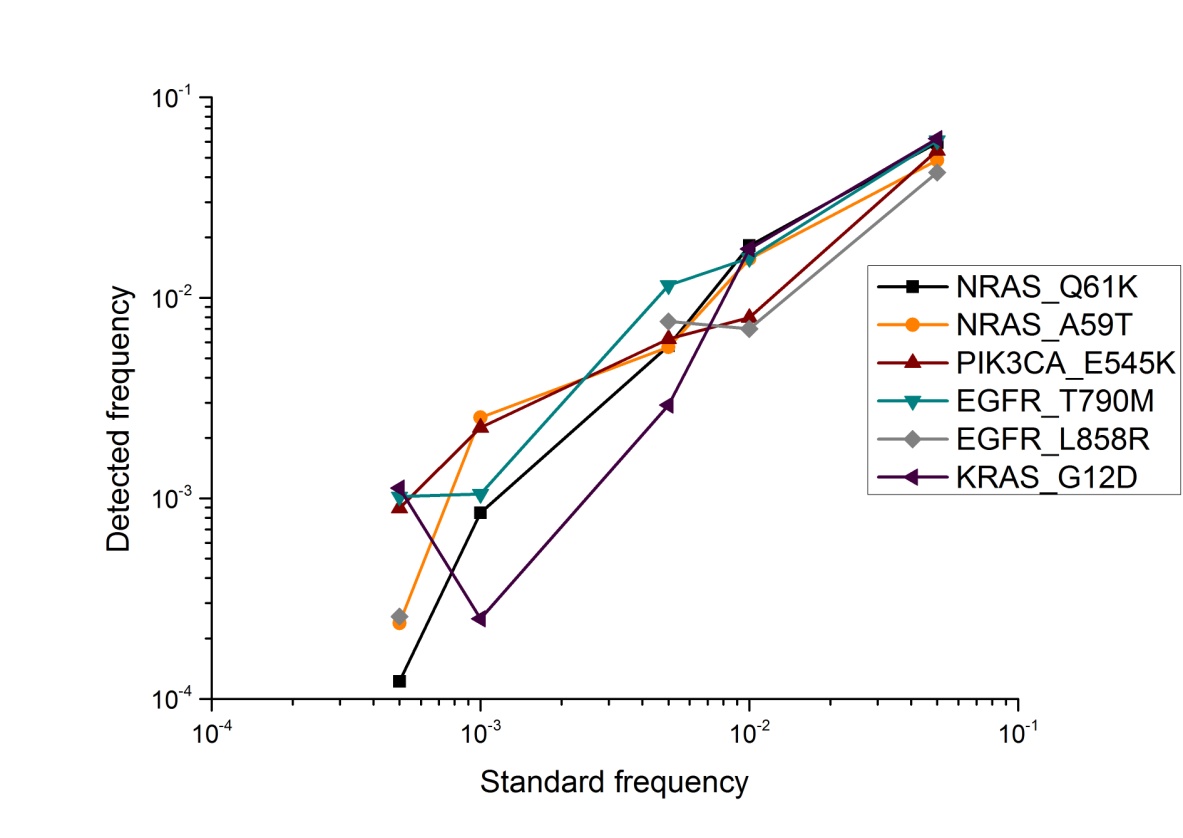


**Figure S1** Sensitivity estimation using reference ctDNA standards. Six references genes were used as the standards. The x-axis is the frequency of spiked DNA in the solution of wild-type DNA standards. The y-axis is the detected frequency as observed from our experiments. Each coloured line represents one hotspot in a cancer gene; the genes are listed in the legend on the right of the figure.

| 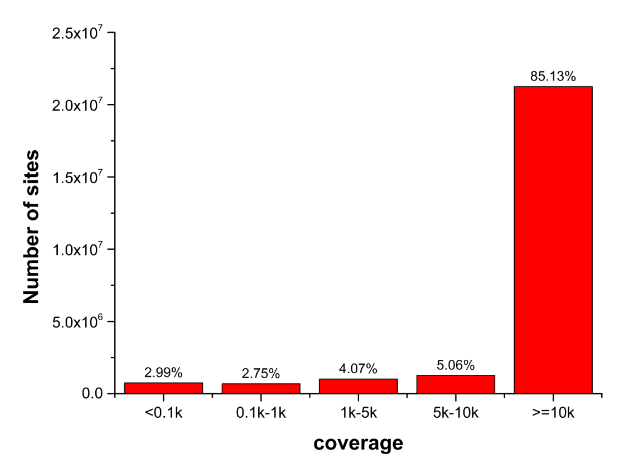 |
| --- |

**Figure S2** Sequence depth analysis of the 1134 samples. The majority of sites were covered for at least 10000×.

**
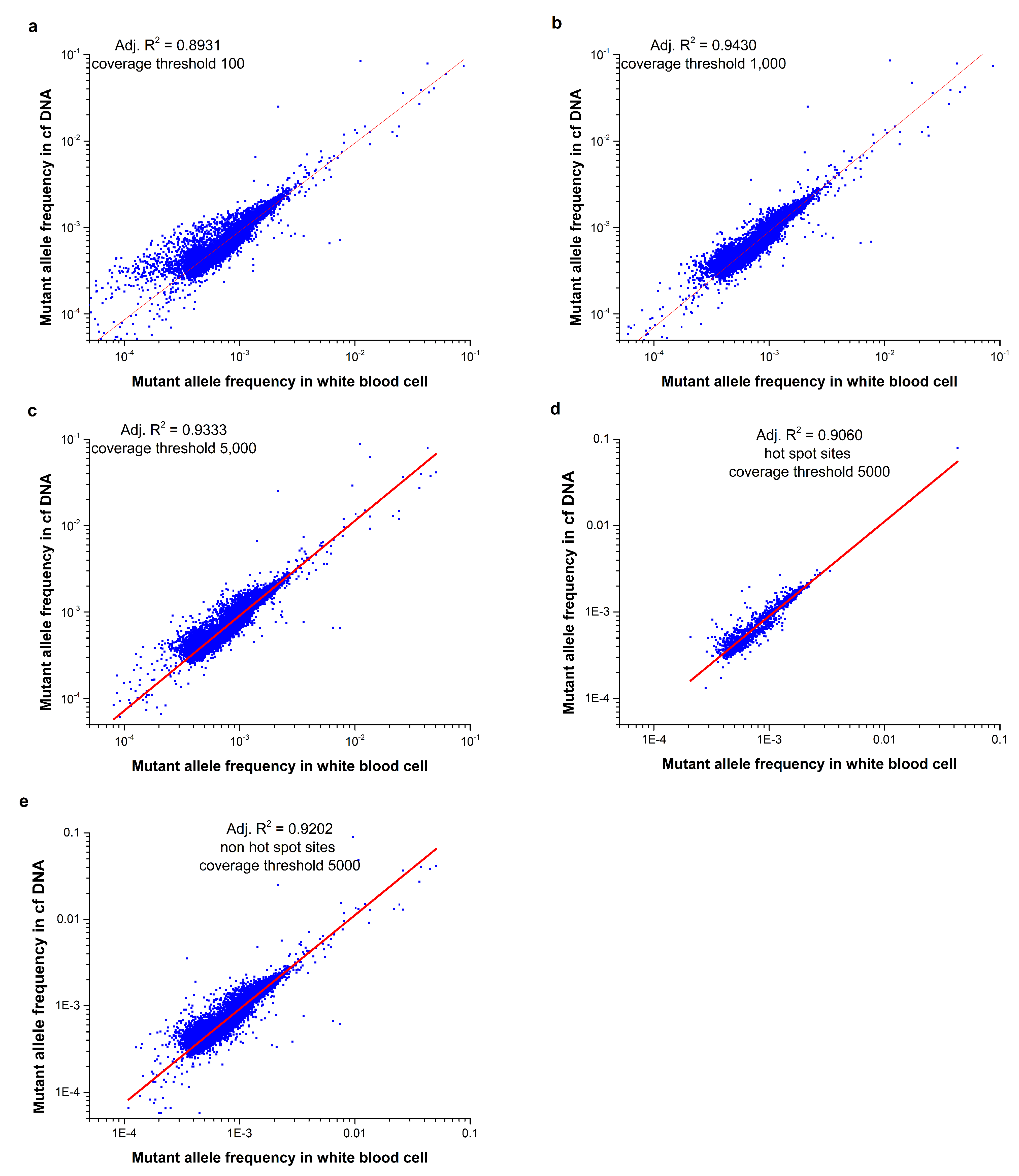
**

**Figure S3** Correlation study of mutant allele frequency in WBC and cfDNA. The correlations at different minimal sequence depth, 100× (a), 100× (b) and 5000× (c), and at different positions, hotspot (d) and non-hot spot sites (e).

**
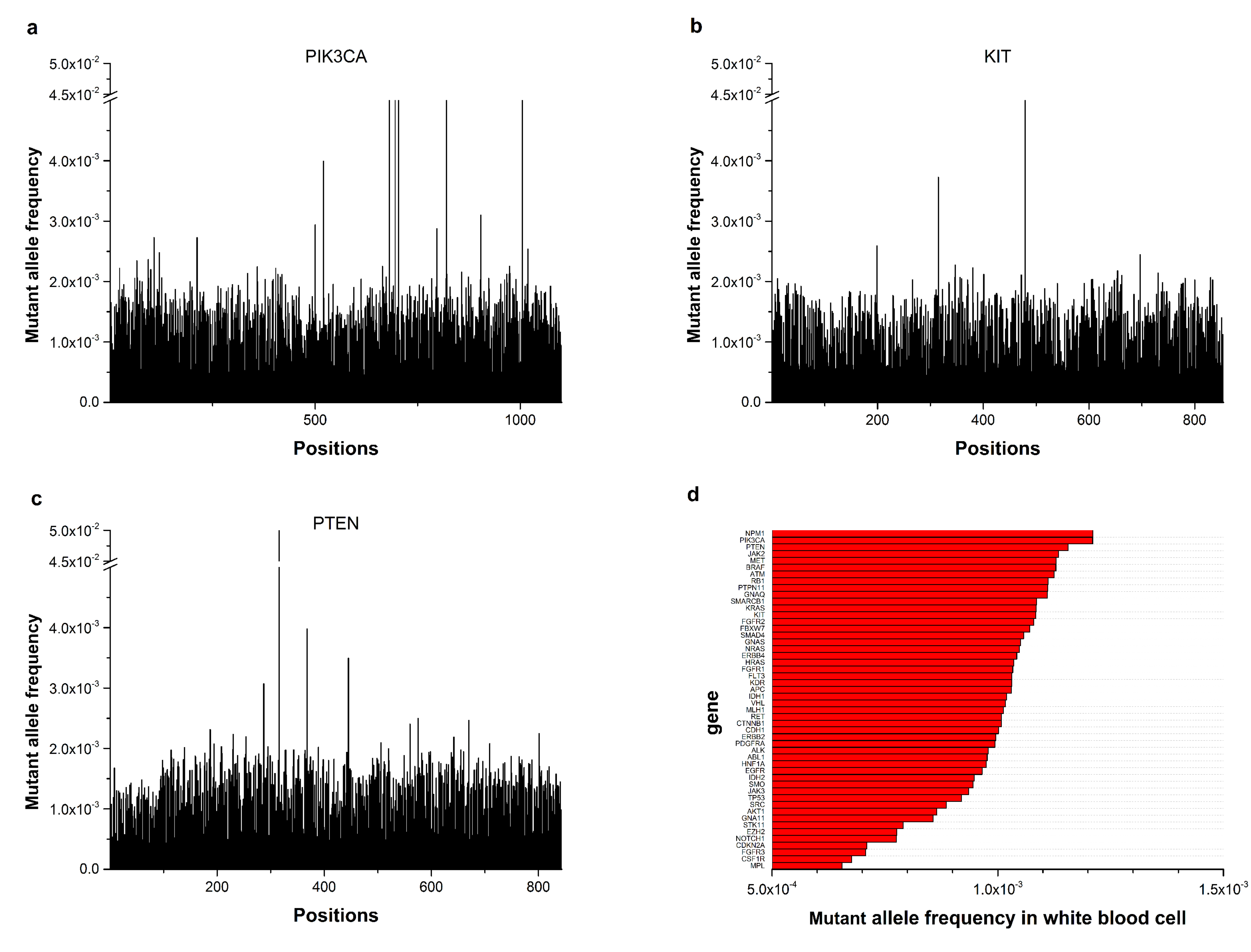
**

**Figure S4. Ranking of average mutant allele frequency of 50 cancer-associated genes**

The mutant allele frequency of *PIK3CA* gene (a), *KIT* gene (b) and *PTEN* gene (c). (d) Mutant allele frequency of 50 genes in WBC. The positions included in this analysis are 22027 nucleotide positions with a sequencing depth higher than 10000× from 309 WBC samples in which we have the corresponding cfDNA data. The positions are sorted by their gene names and classified back into 50 genes.


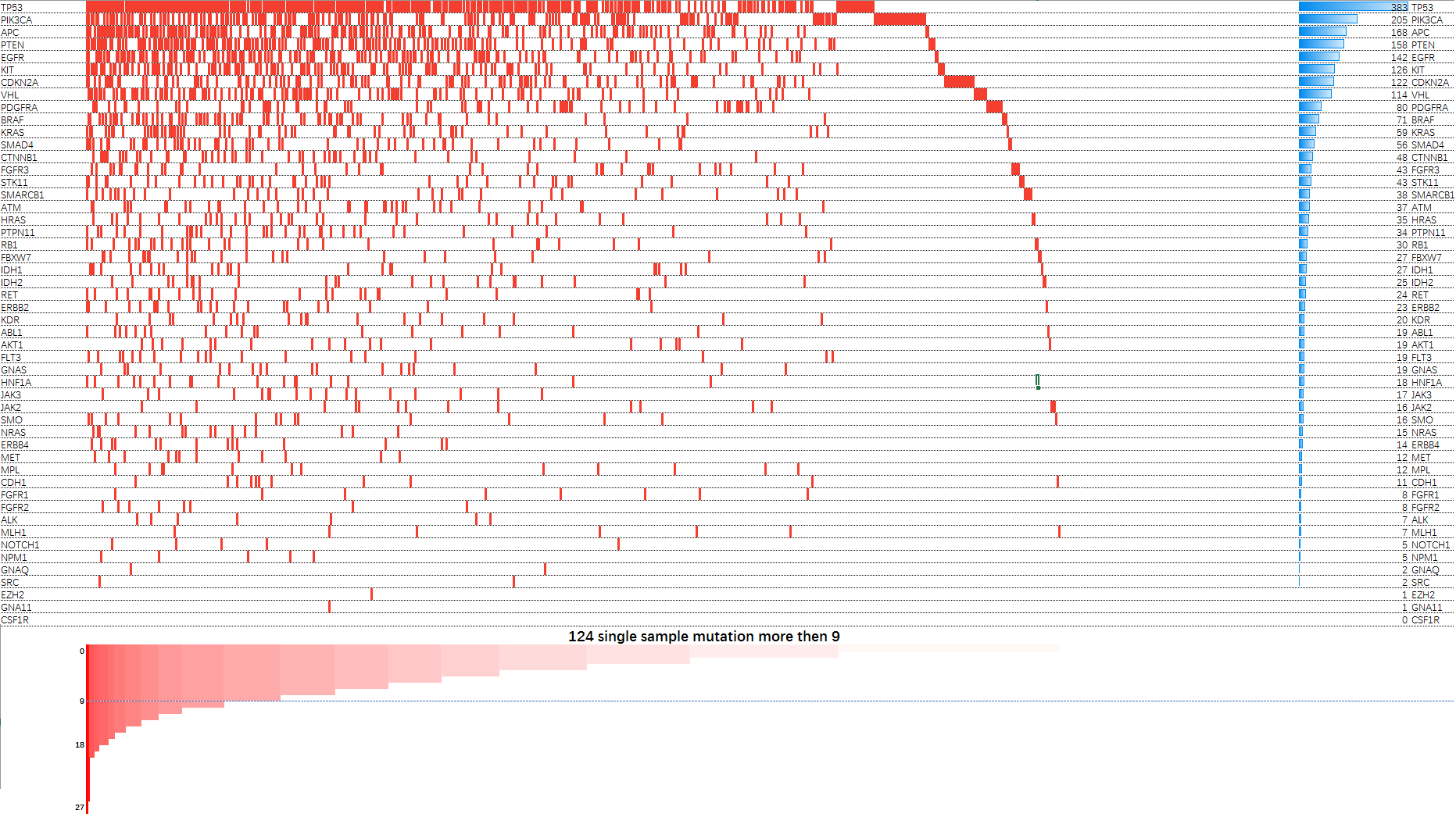


**Figure S5. Spectrum of hotspot-mutations profile in 775 cfDNA samples.** The columns are 775 samples, and 50 rows represent 50 cancer-associated genes. We define the mutation as the variant allele frequency larger than 1% and the average depth more than 5000x (For demonstration). The red box indicates at least one mutation is detected in that gene of that patient. The histogram on the right panel is the number of mutations discovered in each gene, and histogram in the bottom panel shows the number of discovered mutation gene in each sample.
